# Calcium, Magnesium, and Zinc Supplementation during Pregnancy: The Additive Value of Micronutrients on Maternal Immune Response after SARS-CoV-2 Infection

**DOI:** 10.3390/nu14071445

**Published:** 2022-03-30

**Authors:** Ioana Mihaela Citu, Cosmin Citu, Madalin-Marius Margan, Marius Craina, Radu Neamtu, Oana Maria Gorun, Bogdan Burlea, Felix Bratosin, Ovidiu Rosca, Mirela Loredana Grigoras, Andrei Motoc, Daniel Malita, Octavian Neagoe, Florin Gorun

**Affiliations:** 1 Department of Internal Medicine I, “Victor Babes” University of Medicine and Pharmacy Timisoara, 300041 Timisoara, Romania; citu.ioana@umft.ro; 2 Department of Obstetrics and Gynecology, “Victor Babes” University of Medicine and Pharmacy Timisoara, 300041 Timisoara, Romania; marganmm@gmail.com (M.-M.M.); crainamariuslucian@gmail.com (M.C.); radu.neamtu@umft.ro (R.N.); gorun.florin@umft.ro (F.G.); 3 Department of Obstetrics and Gynecology, Municipal Emergency Clinical Hospital Timisoara, 300202 Timisoara, Romania; oanabalan@hotmail.com (O.M.G.); bogdanburlea@yahoo.com (B.B.); 4 Methodological and Infectious Diseases Research Center, Department of Infectious Diseases, “Victor Babes” University of Medicine and Pharmacy, 300041 Timisoara, Romania; felix.bratosin7@gmail.com (F.B.); ovidiu.rosca@umft.ro (O.R.); 5 Department of Anatomy and Embryology, “Victor Babes” University of Medicine and Pharmacy Timisoara, 300041 Timisoara, Romania; grigoras.mirela@umft.ro (M.L.G.); amotoc@umft.ro (A.M.); 6 Department of Radiology, “Victor Babes” University of Medicine and Pharmacy Timisoara, 300041 Timisoara, Romania; malita.daniel@umft.ro; 7 First Department of Surgery, Second Discipline of Surgical Semiology, “Victor Babes” University of Medicine and Pharmacy, 300041 Timisoara, Romania; dr.octavian.neagoe@gmail.com

**Keywords:** COVID-19, SARS-CoV-2, pregnancy, micronutrients, nutritional supplementation

## Abstract

Magnesium may contribute to the immune response during and after SARS-CoV-2 infection by acting as a cofactor for immunoglobulin production and other processes required for T and B cell activity. Considering magnesium as a recommended dietary supplement during pregnancy and the possible role of magnesium deficiency in COVID-19 and its complications, the current study sought to determine the effect of magnesium and magnesium-containing nutritional supplements on the immune response following SARS-CoV-2 infection in pregnant women, as well as to observe differences in pregnancy outcomes based on the supplements taken during pregnancy. The study followed a cross-sectional design, where patients with a history of SARS-CoV-2 infection during their pregnancy were surveyed for their preferences in nutritional supplementation and their profile compared with existing records from the institutional database. A cohort of 448 pregnant women with COVID-19 during 22 months of the pandemic was assembled, out of which 13.6% took a magnesium-only supplement, and 16.5% supplemented their diet with a combination of calcium, magnesium, and zinc. Around 60% of patients in the no-supplementation group had the SARS-CoV-2 anti-RBD lower than 500 U/mL, compared with 50% in those who took magnesium-based supplements. A quantity of magnesium >450 mg in the taken supplements determined higher levels of antibody titers after COVID-19. Low magnesium dosage (<450 mg) was an independent risk factor for a weak immune response (OR-1.25, *p*-value = 0.003). The observed findings suggest supplementing the nutritional intake of pregnant women with magnesium-based supplements to determine higher levels of SARS-CoV-2 anti-RBD antibodies, although causality remains unclear.

## 1. Introduction

Since the SARS-CoV-2 outbreak, substantial research has been conducted to determine its mechanism of action in order to create an efficient antiviral treatment and vaccine to combat the SARS-CoV-2 infection and its severe manifestations [1,2,3,4,5,6,7]. Though effective vaccines have been used in a global immunization program against COVID-19 since early 2021 [8,9], some promising antiviral medications are just being released [10], although their access and distribution are limited, and there is an existent reluctancy in administering them to pregnant women. Additionally, apart from immunization, there are other unanswered questions about putative protective factors against severe forms of COVID-19 in pregnant women, since their pregnancy status increases the risk of infections [11,12,13]. Due to the increased risk of COVID-19 severity and complications in pregnant women, it is necessary to understand what puts them at risk and what is likely to protect them [14,15]. It was recently promoted that lactoferrin is a useful supplement during viral infections [16], therefore, supplementing it during pregnancy can aid in preventing severe SARS-CoV-2 infections and negative outcomes.

The immune system needs enough and proper nourishment to operate efficiently [17,18,19]. During an active illness, such as the SARS-CoV-2 infection, the work of the immune system boosts the need for energy even more, with increased baseline energy expenditure. Thus, an optimum diet for the greatest immunological results would be nutrition that supports immune cells’ functioning, enabling them to launch efficient responses against pathogens but also to terminate the reaction promptly when required, avoiding any underlying persistent inflammation. The immune system′s energy and food requirements may be satisfied by exogenous sources such as diet alone, or through endogenous sources such as body storage if dietary sources are insufficient. Certain micronutrients and dietary components play critical roles in the formation and maintenance of a functional immune system throughout the course of a person′s life, as well as in the reduction of chronic inflammation [20]. For example, zinc (Zn) is a necessary micronutrient for cell proliferation, differentiation, RNA and DNA synthesis, as well as cell structure and membrane stability. Additionally, there is compelling evidence linking zinc deficiency to a number of infectious disorders. Zinc is also involved in the regulation of proinflammatory cytokines and in the management of oxidative stress [21,22].

Apart from zinc, iron deficiency during pregnancy was proven a risk factor for negative outcomes [23]. Anemia was more prevalent in pregnant women infected with SARS-CoV-2, and illness severity was worse in pregnancies diagnosed with iron deficiency anemia. The neonatal birth weight was also significantly lower in pregnancies associated with iron deficiency [24].

Magnesium is the fourth most abundant mineral in the human body, behind calcium, sodium, and potassium, and the second most abundant intracellular cation following potassium [25]. As a micronutrient, it is involved in multiple functions of the human organism, including immunity. Magnesium is found predominantly inside cells, where it functions as a counter ion to the energetic ATP and nuclear acids [26]. Magnesium is a cofactor in over 600 enzyme systems in the body that govern a variety of physiological activities, including protein synthesis, muscle and nerve transmission, neuromuscular conduction, signal transduction, blood glucose management, and blood pressure regulation [27,28]. Magnesium is also required for the active transport of calcium and potassium ions across cell membranes, which is required for nerve impulse transmission, muscular contraction, vasomotor tone, and proper heart rhythm. The recommended daily zinc supplementation in pregnant women is around 100 mg [29].

The consumption of magnesium remains below recommended levels in Europe and the United States, according to dietary surveys. The population of industrialized nations consumes contemporary fast-food diets that are deficient in magnesium. According to several research, these diets include between 30% and 50% less magnesium than the daily recommended dosage [30]. It is claimed that magnesium intakes in the United States have decreased over the previous century, from around 500 mg/day to 175–225 mg/day [31]. The recommended daily magnesium intake in the general adult population ranges between 240 and 420 mg [32,33], whereas pregnant women have a recommended daily magnesium intake of 350–400 mg [34]. Therefore, we developed a study aiming to observe the impact of magnesium and magnesium-containing nutritional supplements on the immune response after SARS-CoV-2 infection in pregnant women as the main end-point, while also trying to determine evidence of pregnancy outcomes and fetal development as secondary targets of the study.

## 2. Materials and Methods

### 2.1. Study Design and Ethical Considerations

The current study followed a cross-sectional design following pregnant women who presented in the outpatient setting of the Obstetrics and Gynecology Clinic of the Timisoara Municipal Emergency Hospital affiliated with the University of Medicine and Pharmacy from Timisoara, Romania during a 22-month period, from 14 April 2020 to 14 February 2022. Patients were notified about the study′s purpose and significance and were required to sign an informed consent form after all their questions were addressed. Our research followed the Helsinki Declaration′s guidelines for conducting scientific studies involving human participants, and it was authorized by the Timisoara Municipal Hospital′s Scientific Ethics Committee on 29 December 2021, with the approval number I-32885.

### 2.2. Inclusion Criteria and Study Variables

All patients were reached and surveyed online in accordance with the recommendations for social distancing during the COVID-19 pandemic. The purpose of the questionnaire was to determine the use of nutritional supplements containing magnesium, or the combination between calcium, magnesium, and zinc during pregnancy. The main questions in the survey included the type of supplement used, the duration of use, the pattern of administration, and the dosage. Other relevant clinical data were obtained by trained medical personnel participating in the study from the patients’ records, in accordance with the patient consent form. A convenience sampling method was utilized to calculate the ideal sample size, which was determined to be at least 381 pregnant women, for a 5% margin of error at a 95% level of confidence. The inclusion criteria comprised the status of giving birth within six months before survey completion, having an RT-PCR confirmed SARS-CoV-2 infection before giving birth, not receiving a COVID-19 vaccine, and having the result from a SARS-CoV-2 antibody check at the time of hospital admission for labor onset. Women with documented renal illness, autoimmune disease, type 1 diabetes mellitus, or those taking immunosuppressive medications were excluded from the study. At the end of the study period, a total of 448 pregnant females met the inclusion criteria and satisfied the adequate sample size.

The variables collected for statistical analysis comprised the background data of pregnant women (age, gravidity, parity, area of residence, occupation, level of education, level of income, and civil status), summary of nutritional supplementation (reason for supplementation, duration of supplementation, other supplements taken, consistency of intake, form of magnesium in the supplements, and quantity of magnesium), clinical characteristics, outcomes and complications (comorbidities, COVID-19 severity, maternal complications, neonatal complications, birth weight, and APGAR score), and SARS-CoV-2 IgG antibody levels at birth.

### 2.3. Statistical Analysis

The statistical analysis was performed with IBM SPSS v.26 (SPSS Inc., IBM Corp. Armonk, NY, USA) and MedCalc v.20 (MedCalc Software Ltd., Ostend, Belgium). We computed the absolute and relative frequencies of categorical variables. For comparison of proportions, we used the Chi-square and Fisher’s tests, and for comparison of group differences in nonparametric data, the Mann–Whitney or Kruskal–Wallis tests were utilized. Risk analysis for weak immune response was performed by using a logistic regression model, and risk factors were reported as odds ratios (ORs) with 95% confidence intervals (CIs). The significance threshold was set for an alpha value of 0.05.

## 3. Results

### 3.1. Background Analysis

From the total of 448 women included in the study, the vast majority of 313 (69.9%) did not follow a specific diet supplementation during their pregnancy, whereas 61 (13.6%) took a magnesium-only supplement, and the remaining 74 (16.5%) supplemented their diet with a combination of calcium, magnesium, and zinc. The background data of the study cohort presented in Table 1 indicated many significant disparities between the three study groups. The participants who supplemented their diets with magnesium-based products were in higher proportions at their first gravidity and parity compared with those who did not take Mg supplements (62.3% vs. 62.2% vs. 41.2%, *p*-value < 0.001), respectively (67.2% vs. 66.2% vs. 43.8%, *p*-value < 0.001). A statistically significant difference was also observed between patients who supplemented their diets and those who did not in what regards their area of residence and occupation. It was determined that pregnant women who did not take nutritional supplements were in higher proportions living in rural areas and more likely to have no occupation, as compared with those who followed a magnesium-based dietary supplement. A total of 55.6% pregnant women with no supplementation were living in rural areas, versus 37.7% women who took magnesium-only supplements and 40.5% women who took calcium, magnesium, and zinc supplements (*p*-value = 0.006). Moreover, 19.8% pregnant women in the no supplementation group were unemployed, compared with 13.1% women who took magnesium-only supplements and 14.9% women who took calcium, magnesium, and zinc supplements (*p*-value = 0.028). As a consequence, the level of income of those in the no supplementation group was significantly lower than that of the other two groups (*p*-value = 0.042).

Table 2 describes the observed findings with regards to nutritional supplementation behavior in the study cohort during pregnancy. There were no statistically significant differences between the group of women who took magnesium-only supplements and the group with calcium, magnesium, and zinc supplementation. However, it was observed that the majority of pregnant women decided to take magnesium supplements by self-belief (63.9% vs. 59.5%), and less than 30% in both groups followed it strictly for nine months throughout their pregnancy. Around 70% of all pregnant women also supplemented their diets with iron (67.2% vs. 70.3%), followed by folate and vitamin D. Over 60% of pregnant women who took magnesium supplements were consistent with daily use, and the most frequent dose of magnesium was higher than 450 mg (67.2% s—magnesium-only vs. 64.9%—Ca+Mg+Zn). Magnesium citrate was the most common form of magnesium in the composition of supplements taken by pregnant women (34.4% vs. 36.6%), followed by magnesium salts (MgO) used by more than 20% of study participants, and magnesium aspartate (13.1% vs. 14.1%).

### 3.2. Analysis of Magnesium Supplementation Effects

An in-depth analysis of clinical characteristics, complications, and outcomes of the study groups presented in Table 3 did not find any significant variations in comorbidities suffered by study participants during their pregnancy. COVID-19 severity was mild in more than 70% of cases, without important differences in proportions between study groups (*p*-value = 0.868). The most frequent maternal complication was anemia, affecting about one third of all participants; however, the premature rupture of membranes (PROM) was statistically significantly higher in frequency in the group of pregnant women with no nutritional supplementation after SARS-CoV-2 infection (10.9% vs. 3.3% vs. 4.1%, *p*-value = 0.047). A big but not significant difference was discovered in the proportions of gestational hypertension between study groups. Although the comparison within groups was not statistically significant (8.0% vs. 3.3% vs. 2.7%, *p*-value = 0.138), the analysis between groups showed a significant difference in proportions of pregnant women with gestational hypertension who did not take nutritional supplements, compared with pregnant women who took a calcium, magnesium, and zinc supplement during pregnancy (8.0% vs. 2.7%, *p*-value = 0.044).

Although maternal complications were not very common, it was observed that neonatal complications were more likely to occur. There was a statistically significantly higher proportion of premature births in the group of COVID-19 pregnant women who did not supplement their diet compared with those who took magnesium supplements (14.4% vs. 6.6% vs. 5.4%, *p*-value = 0.038). In consequence with the higher incidence of premature births in the group of patients without nutritional supplementation, the birth weight and APGAR scores were also significantly lower than in the newborns of mothers who took magnesium-based supplements. There were 41 (13.1%) newborns with a birth weight between 1500 g and 2000 g in the no-supplementation group, compared with 3 (4.9%), and 3 (4.1%) in the groups of magnesium supplements, respectively (*p*-value = 0.037). Furthermore, 57 newborns were given an APGAR score between 7 and 8 in the group of mothers who did not follow a magnesium-based nutritional supplementation, compared with 4 (6.6%) and 6 (8.1%) in the other groups (*p*-value = 0.033). Lastly, an important difference in immune response after clearing the SARS-CoV-2 infection was observed between study groups. Therefore, only 37.1% of mothers from the no-supplementation group had a level of SARS-CoV-2 anti-RBD higher than 500 U/mL, compared with 50.8% in the group of magnesium-only supplementation, and 48.6% in those who took a combination of calcium, magnesium, and zinc (*p*-value = 0.044) (Table 3).

Other differences between study groups were observed in the analysis of COVID-19 symptoms experienced by pregnant women during their pregnancy (Table 3). Mothers who did not take magnesium-based nutritional supplements had higher proportions of symptoms such as anosmia/ageusia, fatigue, and digestive problems. A total of 179 (57.2%) women in the no-supplement group reported having anosmia or ageusia, compared with 27 (44.3%) and 31 (41.9%) in those who took magnesium-based supplements, respectively (*p*-value = 0.020). Moreover, there were 272 (86.9%) pregnant women in the no-supplement group complaining of fatigue during SARS-CoV-2 infection, compared with 46 (75.4%) and 54 (73.0%) women who took supplements, respectively. Lastly, an important difference in symptom prevalence was seen in digestive symptoms, affecting 28.1% women who did not take magnesium-based supplements, compared with 18.0% and 16.2% in those who followed a nutritional supplementation.

**Figure 1:**
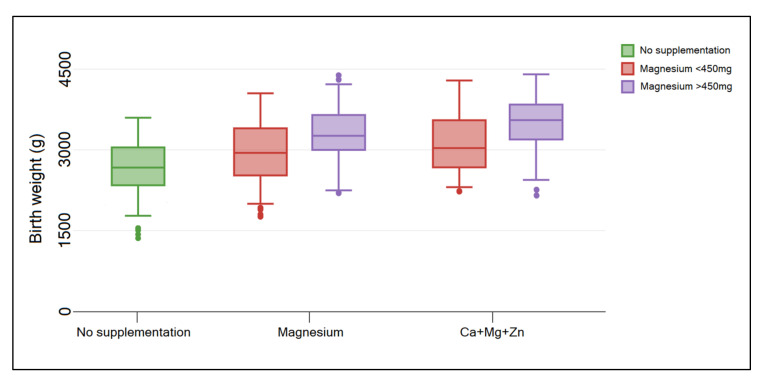
Box-plot of newborn birth weight from mothers with COVID-19 during pregnancy, stratified by nutritional supplementation with magnesium-based supplements and magnesium dose. Data analyzed by Kruskal–Wallis test.

**Figure 2:**
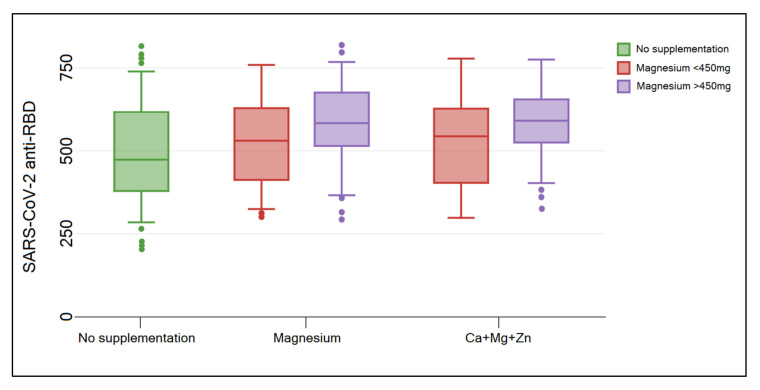
Box-plot of maternal immune response after SARS-CoV-2 infection clearance, measured during labor and stratified by nutritional supplementation with magnesium-based supplements and magnesium dose. Data analyzed by Kruskal–Wallis test.

The box-plot analysis presented in Figure 1 and Figure 2 identified a significant difference in birth weight of newborns from mothers with COVID-19 who did not follow a nutritional supplementation with magnesium-based products during pregnancy, compared with those who took magnesium supplements (*p*-value = 0.022). There was no significant difference within groups of magnesium-only and Ca+Mg+Zn. A similar finding was determined in the immune response, where the SARS-CoV-2 anti-RBD titers of women who supplemented their diet were significantly higher than in the group with no supplements (*p*-value = 0.041). There was no significant difference within groups of magnesium-only and Ca+Mg+Zn. Furthermore, a significant contributor was the dose of magnesium taken, where a bigger birth weight and higher SARS-CoV-2 anti-RBD levels were determined by a magnesium dose higher than 450 mg.

The multivariate analysis presented in Table 4 determined the multiple independent risk factors associated with a weak immune response, stratified by nutritional supplementation status. In the no supplementation group, it was observed that age older than 34 years (OR-1.12, *p*-value = 0.047), obesity (OR-1.56, *p*-value = 0.011), and a duration of more than 90 days since COVID-19 clearance (OR-1.62, *p*-value = 0.001) were independent risk factors for a weak immune response. In the magnesium supplementation group, only obesity (OR-1.42, *p*-value = 0.040), low magnesium dosage (OR-1.25, *p*-value = 0.003), and a duration of more than 90 days since COVID-19 clearance (OR-1.27, *p*-value = 0.008) were determined as significant risk factors for a weak immune response.

## 4. Discussion

The current study achieved its main goal of studying the impact of magnesium and magnesium-containing nutritional supplements on immune response after SARS-CoV-2 infection in pregnant women and determined several important differences in pregnancy outcomes in the same patients, based on the supplements taken during pregnancy. The most important finding is the observed weaker immune response in pregnant women who did not supplement their diet with magnesium-based products. Around 60% of patients in this group had the SARS-CoV-2 anti-RBD lower than 500 U/mL, compared with 50% in those who took nutritional supplementation. Furthermore, the quantity of magnesium in the taken supplements should be higher than 450 mg to determine a higher level of antibody titers after COVID-19. Similar findings were determined on newborn birth weight based on the type of nutritional supplementation and the magnesium dose. Pregnant women infected with SARS-CoV-2 during their pregnancy who did not take any magnesium supplementation were more likely to give birth to newborns weighting significantly less than those born in the groups who followed a diet.

Different observational studies assessed the influence magnesium, as a micronutrient, has on COVID-19 pathogenesis, prognosis, and immune reaction after infection clearance. A review of literature studied the nutritional factors that seem to have a role in the development and prognosis of COVID-19 [35]. Obesity, a condition linked with insufficient magnesium intake, was found to be a risk factor for increased mortality and hospitalizations in COVID-19 patients. Additionally, magnesium is a cofactor required for the production, transport, and activation of vitamin D, and both magnesium and vitamin D deficiency have been linked to a number of chronic disorders. According to several findings, magnesium and vitamin D deficiency seem to have a role in the etiology of COVID-19 [36]. COVID-19 is associated with significant lung and cardiac dysfunction, and magnesium is required for proper lung and heart function. Along with obesity, other coexisting conditions such as high blood pressure, diabetes mellitus and, most significantly, advanced age are associated with increased COVID-19 severity, possibly due to an underlying chronic inflammatory state or a lower threshold for the development of organ dysfunction as a result of the immune response [37,38,39]. All of the above-mentioned illnesses, as well as old age and a chronic inflammatory state, have been linked to a deficient magnesium level.

Other studies documented the importance of zinc as a requirement for the pathogenesis of viral infections and evidence of a link between zinc levels and the severity of illnesses. Anosmia, a typical COVID-19 symptom, was also described as present in zinc insufficiency [40]. Zinc supplements added to the chloroquine treatment regimen improved the antiviral mode of action and treatment effectiveness in COVID-19 patients [41], although this medication proved inefficient for the different SARS-CoV-2 strains. Zinc may provide protective benefits, according to another research, by lowering inflammation, increasing mucociliary clearance, avoiding ventilator-induced lung damage, and modifying antiviral and antibacterial immunity [42,43]. As a result, zinc can be utilized as a prophylactic measure as well as an adjuvant treatment for COVID-19. Besides zinc intake, other studies suggested supplementing pregnant women with iron and selenium, which boosted the immune system during pregnancy and proved to efficiently prevent complications in COVID-19-infected pregnant women [44]. The serum selenium level fell progressively during pregnancy, but this normal decline was accelerated by the COVID-19 infection. The explanation for this might be that higher selenium requirements are dependent on the immunological response to illness. Reduced maternal selenium levels were observed to be associated with increased IL-6 and D-dimer levels, indicating selenium’s involvement in illness development [45].

Our findings documenting the immune response in the cohort of pregnant women with a cleared SARS-CoV-2 infection during their pregnancy is consistent with a research that analyzed the SARS-CoV-2 anti-RBD levels in COVID-19 confirmed patients after infection clearance in the general population. It was observed that anti-RBD antibodies were detected at levels more than 250 U/mL in 44% of people with confirmed COVID-19 infection and at levels greater than 500 U/mL in only 27% of samples analyzed. However, our research differed in that the median number of months after COVID-19 diagnosis was 8.7 months, while 40% of our patients had COVID-19 within less than 3 months. There was no correlation between the time period after infection and antibody titer [46].

Since the patients were surveyed about their past use of nutritional supplements during pregnancy, including the dosage and type of supplement, recall bias is an important limitation that can alter the observed results of this study and affect data reproducibility. Another limitation is the lack of micronutrient analysis in the serum of pregnant women with SARS-CoV-2 infection, to determine if they were lower than the cut-off values. It is also worth noting that our research lacked direct neutralization testing and that antibody levels alone do not directly correlate with individuals′ degree of immunity.

## 5. Conclusions

Pregnant women who supplemented their diet with calcium, zinc, and magnesium, or magnesium only did not have a different clinical course of disease during the SARS-CoV-2 infection, but they showed a significantly higher titer of SARS-CoV-2 anti-RBD after the infection was cleared. The low serum concentration, or no supplementation of these micronutrients during pregnancy may lead to a weaker immune status. Although the observed findings may suggest supplementing the nutritional intake of pregnant women with calcium, magnesium, and zinc, causality cannot be determined from the circumstances under which the study was developed. However, further prospective research is required to determine their influence on COVID-19.

## Figures and Tables

**Table 1:** Background data of the study cohort stratified by type of micronutrient supplementation.

Variables *	No Supplementation (*n* = 313)	Mg Supplementation (*n* = 61)	Ca+Mg+Zn Supplementation (*n* = 74)	*p*-Value
Age				0.298
<25	34 (10.9%)	9 (14.8%)	13 (17.6%)	
25–34	221 (70.6%)	40 (65.6%)	43 (58.1%)	
>34	58 (18.5%)	12 (19.7%)	18 (24.3%)	
Gravidity				<0.001
1	129 (41.2%)	38 (62.3%)	46 (62.2%)	
2	94 (30.0%)	14 (23.0%)	22 (29.7%)	
≥3	90 (28.8%)	9 (14.8%)	6 (8.1%)	
Parity				<0.001
1	137 (43.8%)	41 (67.2%)	49 (66.2%)	
2	102 (32.6%)	16 (26.2%)	20 (27.0%)	
≥3	74 (23.6%)	4 (6.6%)	5 (6.8%)	
Area of residence				0.006
Urban	139 (44.4%)	38 (62.3%)	44 (59.5%)	
Rural	174 (55.6%)	23 (37.7%)	30 (40.5%)	
Occupation				0.028
No occupation	62 (19.8%)	8 (13.1%)	11 (14.9%)	
Student	54 (17.3%)	17 (27.9%)	24 (32.4%)	
Employed	197 (62.9%)	36 (59.0%)	39 (52.7%)	
Level of education				0.156
Elementary	38 (12.1%)	4 (6.6%)	7 (9.5%)	
Middle	106 (33.9%)	15 (24.6%)	19 (25.7%)	
Higher	169 (54.0%)	42 (68.9%)	48 (64.9%)	
Level of income				0.042
Low	63 (20.1%)	9 (14.8%)	10 (13.5%)	
Medium	195 (62.3%)	34 (55.7%)	41 (55.4%)	
High	55 (17.6%)	18 (29.5%)	23 (31.1%)	
Civil status				0.923
Married	283 (90.4%)	57 (93.4%)	66 (89.2%)	
Single	12 (3.8%)	2 (3.3%)	3 (4.1%)	
Divorced/Widowed	18 (5.8%)	2 (3.3%)	5 (6.8%)	

* Data presented as *n* (frequency) unless specified differently.

**Table 2:** Summary of nutritional supplementation in the study cohort, stratified by type of micronutrient supplementation.

Variables *	Mg Supplementation (*n* = 61)	Ca+Mg+Zn Supplementation (*n* = 74)	*p*-Value
Reason for supplementation			0.594
Self-medicated	39 (63.9%)	44 (59.5%)	
By recommendation	22 (36.1%)	30 (40.5%)	
Duration of supplementation			0.266
<9 months	48 (78.7%)	52 (70.3%)	
≥9 months	13 (21.3%)	22 (29.7%)	
Other supplements used			
Folate	24 (39.3%)	39 (52.7%)	0.121
Iron	41 (67.2%)	52 (70.3%)	0.702
Vitamin D	18 (29.5%)	24 (32.4%)	0.714
Probiotics	14 (23.0%)	21 (28.4%)	0.473
Consistency of intake			0.852
Daily	39 (63.9%)	45 (60.8%)	
At least 5 times a week	18 (29.5%)	25 (33.8%)	
Less than 5 times a week	4 (6.6%)	4 (5.4%)	
Form of magnesium			0.967
Mg(OH)_2_	6 (9.8%)	6 (8.5%)	
MgO	13 (21.3%)	16 (22.5%)	
MgCl_2_	7 (11.5%)	5 (7.0%)	
Lactate	6 (9.8%)	8 (11.3%)	
Citrate	21 (34.4%)	26 (36.6%)	
Aspartate	8 (13.1%)	10 (14.1%)	
Magnesium dose			0.774
<450 mg	20 (32.8%)	26 (35.1%)	
≥450 mg	41 (67.2%)	48 (64.9%)	

* Data presented as *n* (frequency) unless specified differently.

**Table 3:** Clinical characteristics, complications, and outcomes of the study cohort, stratified by type of micronutrient supplementation.

Variables *	No Supplementation (*n* = 313)	Magnesium Supplementation (*n* = 61)	Ca+Mg+Zn Supplementation (*n* = 74)	*p*-Value
Comorbidities				
Obesity, (BMI ≥ 30 kg/m^2^)	65 (20.8%)	11 (18.0%)	14 (18.9%)	0.859
High blood pressure	28 (8.9%)	5 (8.2%)	7 (9.5%)	0.967
Thromboembolic events	7 (2.2%)	0 (0.0%)	1 (1.4%)	0.460
Others **	16 (5.1%)	4 (6.6%)	4 (5.4%)	0.899
COVID-19 severity				0.868
Mild	229 (73.2%)	47 (77.0%)	53 (71.6%)	
Moderate	70 (22.4%)	12 (19.7%)	19 (25.7%)	
Severe	14 (4.5%)	2 (3.3%)	2 (2.7%)	
COVID-19 symptoms				
Fever	246 (78.6%)	45 (73.8%)	53 (71.6%)	0.369
Anosmia/Ageusia	179 (57.2%)	27 (44.3%)	31 (41.9%)	0.020
Cough	215 (68.7%)	39 (63.9%)	42 (56.8%)	0.139
Fatigue	272 (86.9%)	46 (75.4%)	54 (73.0%)	0.003
Dyspnea	124 (39.6%)	22 (36.1%)	25 (33.8%)	0.607
Digestive symptoms	88 (28.1%)	11 (18.0%)	12 (16.2%)	0.043
Others	71 (22.7%)	11 (18.0%)	14 (18.9%)	0.610
Maternal complications				
Anemia	98 (31.3%)	17 (27.9%)	19 (25.7%)	0.592
Gestational diabetes mellitus	27 (8.6%)	3 (4.9%)	5 (6.8%)	0.573
Gestational hypertension	25 (8.0%)	2 (3.3%)	2 (2.7%)	0.138
Oligohydramnios	17 (5.4%)	1 (1.6%)	3 (4.1%)	0.422
Polyhydramnios	15 (4.8%)	2 (3.3%)	1 (1.4%)	0.379
Abnormal presentation	33 (10.5%)	4 (6.6%)	5 (6.8%)	0.433
PROM	34 (10.9%)	2 (3.3%)	3 (4.1%)	0.047
Cesarean delivery	76 (24.3%)	12 (19.7%)	15 (20.3%)	0.611
Neonatal complications				
Anemia	62 (19.8%)	9 (14.8%)	12 (16.2%)	0.555
Puerperal infection	21 (6.7%)	2 (3.3%)	2 (2.7%)	0.281
Premature birth	45 (14.4%)	4 (6.6%)	4 (5.4%)	0.038
Malformations	3 (1.0%)	0 (0.0%)	1 (1.4%)	0.690
NRDS	16 (5.1%)	2 (3.3%)	2 (2.7%)	0.592
Birth weight				0.037
<1500 g	13 (4.2%)	1 (1.6%)	1 (1.4%)	
1500–2500 g	41 (13.1%)	3 (4.9%)	3 (4.1%)	
>2500 g	259 (82.7%)	57 (93.4%)	70 (94.6%)	
APGAR score				0.033
≥9	238 (76.0%)	54 (88.5%)	66 (89.2%)	
7–8	57 (18.2%)	4 (6.6%)	6 (8.1%)	
≤6	18 (5.8%)	3 (4.9%)	2 (2.7%)	
Days since COVID-19 diagnosis				0.898
<90 days	131 (41.9%)	24 (39.3%)	42 (43.2%)	
≥90 days	182 (58.1%)	37 (60.7%)	42 (56.8%)	
SARS-CoV-2 anti-RBD (U/mL)				0.044
<500	197 (62.9%)	30 (49.2%)	38 (51.4%)	
≥500	116 (37.1%)	31 (50.8%)	36 (48.6%)	

* Data presented as *n* (frequency) unless otherwise specified; ** excluding renal illness, autoimmune disease, and diabetes mellitus; BMI—Body Mass Index; APGAR—Appearance, Pulse, Grimace, Activity, and Respiration; NRDS—Neonatal Respiratory Distress Syndrome.

**Table 4:** Analysis of risk factors for weak immune response (<500 SARS-CoV-2 anti-RBD) of pregnant women based on micronutrient supplementation during pregnancy.

	No Supplementation (OR–95% CI)	*p*-Value	Magnesium Supplementation (OR–95% CI)	*p*-Value
Age				
<25 ^	0.91 (0.72–1.10)	0.572	0.84 (0.60–1.13)	0.662
25–34	0.99 (0.76–1.24)	0.384	0.92 (0.84–1.27)	0.513
>34	1.12 (1.01–1.38)	0.047	1.04 (0.82–1.21)	0.296
Obesity				
No ^	0.94 (0.63–1.48)	0.316	0.88 (0.36–1.44)	0.581
Yes	1.56 (1.28–2.34)	0.011	1.42 (1.05–1.93)	0.040
Duration of supplementation				
<9 months	1.18 (0.78–1.69)	0.427	1.04 (0.75–1.28)	0.338
≥9 months ^	1.01 (0.83–1.26)	0.290	0.92 (0.81–1.05)	0.194
Vitamin D supplementation				
No	1.17 (0.91–1.32)	0.522	1.01 (0.88–1.39)	0.194
Yes ^	0.85 (0.36–1.16)	0.314	0.76 (0.62–0.92)	0.086
COVID-19 severity				
Mild ^	1.33 (1.02–0.78)	0.071	1.24 (1.09–1.76)	0.142
Moderate	1.13 (0.93–1.42)	0.461	1.05 (0.83–1.41)	0.510
Severe	0.86 (0.72–0.98)	0.283	0.82 (0.69–1.05)	0.308
Days since COVID-19 clearance				
<90 days ^	1.10 (0.99–0.48)	0.308	0.99 (0.72–0.98)	0.416
≥90 days	1.62 (1.24–2.15)	0.001	1.27 (1.04–1.82)	0.008
Magnesium dose				
<450 mg ^	-	-	1.25 (1.08–1.66)	0.003
≥450 mg	-	-	0.98 (0.63–1.17)	0.372

^^^ Reference category.

## Data Availability

The data presented in this study are available on request from the corresponding author.
